# Mitochondrial Dysfunction and Increased DNA Damage in Vascular Smooth Muscle Cells of Abdominal Aortic Aneurysm (AAA-SMC)

**DOI:** 10.1155/2023/6237960

**Published:** 2023-01-25

**Authors:** Bengi S. Tavris, Andreas S. Peters, Dittmar Böckler, Susanne Dihlmann

**Affiliations:** ^1^Klinik für Gefäßchirurgie und Endovaskuläre Chirurgie, Universitätsklinik Heidelberg, Im Neuenheimer Feld 420, 69120 Heidelberg, Germany; ^2^Vaskuläre Biomaterialbank Heidelberg, Universitätsklinik Heidelberg, Im Neuenheimer Feld 420, 69120 Heidelberg, Germany

## Abstract

There is increasing evidence for enhanced oxidative stress in the vascular wall of abdominal aortic aneurysms (AAA). Mitochondrial damage and dysfunction are hypothesized to be actors in altered production of reactive oxygen species (ROS) and oxidative stress. However, the role of mitochondria and oxidative stress in vascular remodelling and progression of AAA remains uncertain. We here addressed whether mitochondrial dysfunction is persistently increased in vascular smooth muscle cells (VSMCs) isolated from AAA compared to healthy VSMC. AAA-derived VSMC cultures (AAA-SMC, *n* = 10) and normal VSMC cultures derived from healthy donors (*n* = 7) were grown *in vitro* and analysed for four parameters, indicating mitochondrial dysfunction: (i) mitochondrial content and morphology, (ii) ROS production and antioxidative response, (iii) NADP+/NADPH content and ratio, and (iv) DNA damage, in the presence or absence of angiotensin II (AngII). AAA-SMC displayed increased mitochondrial circularity (rounded shape), reduced mitochondrial area, and reduced perimeter, indicating increased fragmentation and dysfunction compared to healthy controls. This was accompanied by significantly increased O_2_^−^ production, reduced NADP+/NADPH levels, a lower antioxidative response (indicated by antioxidative response element- (ARE-) driven luciferase reporter assays), more DNA damage (determined by percentage of *γ*-H2A.X-positive nuclei), and earlier growth arrest in AAA-SMC. Our data suggest that mitochondrial dysfunction and oxidative stress are persistently increased in AAA-SMC, emphasizing their implication in the pathophysiology of AAA.

## 1. Introduction

Vascular smooth muscle cells (VSMCs) are the major cell type in the vascular wall of the blood vessels and participate in both normal vascular function and pathologic processes such as atherosclerosis and aneurysms. They maintain vascular contractility and mechanical integrity of the vessel wall; regulate vascular tone; synthesize extracellular matrix proteins, such as elastin and collagens; and ensure normal vascular repair. For this purpose, VSMCs show a high plasticity and may change their cellular characteristics in response to many acute and chronic stimuli [1].

Within the aortic media, VSMCs ensure the dynamic properties of the aorta, which must withstand the constant mechanical stress of pulsatile blood flow. When the structural integrity of the aortic media is compromised, their VSMCs respond by inadequate or abnormal connective tissue synthesis, cytokine production, and recruitment of inflammatory cells. Aneurysms occur when the capacity of normal repair by VSMC is exhausted. More and more VSMCs change from a contractile to a synthetic phenotype, proliferate, and migrate, which results in thickening of the aortic wall. The increasing diffusion distance from the lumen and the luminal narrowing of the vasa vasorum (i.e., caused by hypertension) cause ischemia of the media, resulting in VSMC loss as well as aortic degenerative changes. In addition, excessive connective tissue degradation by infiltrating leukocytes contributes to progressive weakening of the vessel wall [2, 3].

Abdominal aortic aneurysm (AAA) is the most common form of aneurysmal disease with chronic dilation typically presenting in the infrarenal region. It is positively associated with male sex, advanced age (above 60 years) and smoking habits [4]. In the absence of treatment, the main complication of AAA is rupture, which is life-threatening and still leads to death in up to 50% of the affected patients, despite modern, minimal invasive treatment modalities [5]. The treatment of progressing AAA before rupture comprises open surgical or endovascular strategies, which are associated with high physical stress and morbidity for the patients [6]. Notably, no specific drugs are available so far to decelerate AAA growth or prevent rupture [7], which is why more research is needed to investigate contribution of cellular processes to AAA progression.

According to histological analyses of human AAA samples, leukocytic infiltration, degradation of extracellular matrix, and disruption of VSMC plasticity and function are the main pathophysiological hallmarks of AAA [4]. Moreover, elevated amounts of reactive oxygen species (ROS) and oxidative stress have been described in histological AAA samples [8–11], particularly within the thrombus-covered aneurysm wall [12]. Because overproduction of ROS and oxidative stress are known to induce inflammation, VSMC apoptosis, and degradation of extracellular matrix in the vascular wall [4, 9], they are particularly interesting targets for pharmaceutical interventions to decelerate AAA growth and prevent rupture. However, the dynamics of ROS production and oxidative stress during AAA pathophysiology are incompletely understood.

Recent studies suggest the involvement of mitochondria and mitochondria-derived oxidative stress in the development of AAA. Using an established animal model of angiotensin II- (AngII-) induced aortic aneurysm, mitochondrial stress in macrophages was identified as a driving force of aneurysm growth [13]. In addition, mitochondria in other cell types, i.e., in VSMC and endothelial cells, might also be involved in AAA, because they are known to control numerous physiological processes in the blood vessels. Endothelial cell mitochondria are involved in calcium homeostasis, apoptosis/necrosis, response to cell stress, and regulation of inflammatory signalling pathways [14]. Mitochondria are also involved in cellular signalling pathways in VSMC. Via physiological ROS production, especially H_2_O_2_, they control essential functions of these cells such as proliferation, migration, differentiation to the contractile or synthetic phenotype, and vascular resistance induced by contraction [15].

Notably, we recently demonstrated that mice with a deficiency of the mitochondrial enzyme nicotinamide-nucleotide-transhydrogenase (Nnt) are more susceptible to AngII-induced aortic aneurysms than their Nnt-proficient counterparts. According to our results, the Nnt-deficient VSMC displayed increased oxidative stress, oxidative DNA damage, and a stronger inflammatory phenotype [16]. This led us to speculate that also the mitochondria of aortic VSMC may be important players in AAA growth. Our assumption is supported by further observations. For example, excess ROS production in mitochondria, resulting in vascular oxidative stress, is associated with two of the main risk factors for AAA: aging and smoking [4, 17]. Tobacco smoke contains several reactive oxygen and nitride species and causes indirect ROS production through toxicants, carcinogens, and metals found in the smoke [18]. Both might affect mitochondrial dynamics and function in VSMC of the aneurysm wall. Finally, AngII, a potential contributor to AAA, was recently shown to induce dysregulation of the mitochondrial life cycle, characterized by excess fission relative to fusion of damaged vascular mitochondria [19].

Given the broad functional range of tasks in aortic VSMC, mitochondrial function and its interaction with ROS production in AAA-derived VSMC (AAA-SMC) are promising targets for future therapeutics. Previous studies have analysed oxidative stress by histological analysis in tissues derived from AAA [8, 10–12]. However, mitochondrial life cycle and ROS-induced damage are dynamic processes, which are continuously counterbalanced in living cells. Because AAA tissues undergo ischemic stress when removed from the patients and are immediately fixed by cryopreservation or chemical agents for further analysis, tissue analysis does not allow to investigate mitochondrial function in living VSMC. Therefore and in contrast to previous studies, we here addressed mitochondrial dysfunction and oxidative stress in AAA-SMC cultures versus healthy controls in *in vitro* studies. Our data present evidence that mitochondrial dysfunction and oxidative stress are persistently increased in AAA-SMC when compared with healthy VSMC, which further supports their implication in the pathophysiology of AAA.

## 2. Materials and Methods

### 2.1. Vascular Smooth Muscle Cell (VSMC) Cultures

Human VSMCs derived from male healthy donors (*n* = 5, age 13-68 years) were purchased from PromoCell (Heidelberg, Germany). In addition, primary vascular smooth muscle cells were isolated from healthy carotid arteries (*n* = 2, age 67 and 82). AAA-SMCs were isolated from human abdominal aortic aneurysm biopsies that were obtained during open surgery (*n* = 10, age 49-74). All procedures were performed according to the standard operating procedures of the Vascular Biobank Heidelberg (VBBH), as previously described [20]. Details of the cell cultures are described in supplementary table S1. All patients gave their written informed consent to the study, which was approved by the ethical committee of the University of Heidelberg (S-301/2013 and amendments; S-091/2021). Briefly, aneurysmatic tissue samples were obtained from apparently noncalcified areas of male adult patients undergoing elective open surgery for repair of their AAA. VSMCs were grown in Smooth Muscle Cell Growth Medium 2 (SMC-GM2; PromoCell, Heidelberg, Germany) supplemented with 100 U/ml penicillin and 100 U/ml streptomycin (Thermo Fisher Scientific, Gibco, Germany) at 37°C and 5%CO_2_ in a humidified atmosphere. For subculturing, the cells were split 1 : 2 when reaching 80-90% confluence. The medium was changed twice a week for routine growth independent of splitting. Three to seven cell cultures at passages 5 to 10 were used for each experiment in this study. A few days before and during the experiments, cells were grown without antibiotics.

### 2.2. MitoTracker Staining and Mitochondrial Morphology Analysis

Cells were seeded on cover slips and treated as indicated in the figure legends. After washing, cells were stained with MitoTracker Red CMXRos (Thermo Fisher Scientific, Waltham, USA) at a final concentration of 180 nM in Smooth Muscle Cell Growth Medium 2 at 37°C 5% CO_2_ for 30 minutes prior to fixation with paraformaldehyde as described above. After final washing for three times, ProLong Antifade Gold Mountant with DAPI (Thermo Fisher Scientific, Waltham, USA) was applied to counterstain cell nuclei. Cells were visualized under a fluorescence microscope (Zeiss Axiostar Plus) with 40x objective, and photos were taken for further analysis. Four to six individual cells were captured and analysed per cell line by using the mitochondrial morphology macro for ImageJ designed by Ruben K. Dagda (https://imagejdocu.list.lu/plugin/morphology/mitochondrial_morphology_macro_plug-in/start [21]. For the characterization of mitochondrial dysfunction, fusion, and fission in VSMC, four main parameters were considered: (1) count: number of mitochondrial particles counted; (2) Mito content: average percentage of cytoplasm occupied by mitochondria per cell; (3) average perimeter: average perimeter of mitochondria (in *μ*m); (4) average circularity: average circularity of mitochondria, 1 being a perfect circle and 0 being a perfect line; and (5) mitochondrial fission count (MFC) was calculated using ImageJ data (mitochondria count × 100/mitochondrial area (%area of cell) per cell).

### 2.3. Total DNA Extraction and Quantification of mtDNA Copy Number by Real-Time PCR

Total DNA was isolated from 1 × 10^6^ cells of each culture using DNeasy Blood and Tissue Kit (Qiagen, Hilden, Germany) according to the instructions of the manufacturer. At the final step, DNA was eluted in 100 *μ*l nuclease-free water. Quantification of mtDNA copies/cell was performed as described previously [18, 22]. Briefly, human nicotinamide adenine dinucleotide (reduced) (NADH) dehydrogenase 1 (MTND1) cDNA clone (SC101172, OriGene Technologies, Rockville, MD, USA) was used as a standard for mtDNA copy number. Equivalents of 10^9^, 10^8^, 10^7^, 10^6^, 10^5^, 10^4^, 10^3^, 10^2^, 10^1^, and 10^0^ copies of a plasmid encoding the MTND1 cDNA were amplified by PCR with an ABI StepOne Plus cycler, and a standard curve was generated by plotting the copy numbers against the cycles at a threshold of 0.4. Primer sequences were as follows: forward CGAGCAGTAGCCCAAACAAT and reverse TGTGATAAGGGTGGAGAGGTT. For analysis of the VSMC samples, 1 *μ*l of total DNA extract from each culture was inserted into PCR. Details of the PCR protocol are available upon request. The cycle numbers obtained at a threshold of 0.4 from each sample were converted to total copy number by using the formula derived from the standard curve. The copy number/cell was calculated as follows: *C* = *Q* × *V*_DNA_/*V*_PCR_ × 1/*V*_EXT_, where *C* is the copy number/cell, *Q* is the quantity (total copy number) of DNA determined by the sequence detector in the PCR, *V*_DNA_ is the volume of cell DNA obtained after extraction (100 *μ*l), *V*_PCR_ is the volume of cell DNA solution used for PCR, and *V*_EXT_ is the number of cells used for DNA extraction.

### 2.4. Transfection and Luciferase Reporter Assays

Transfection of VSMC was performed by electroporation, using the Neon transfection system (Thermo Fisher, Scientific, Waltham, USA) as recommended by the manufacturer. Briefly, proliferating VSMCs (passages 4-8) were harvested and resuspended in Buffer R of the Neon transfection system 100 *μ*l kit (Thermo Fisher, Scientific, Waltham, USA) at a density of 10^7^ cells/ml. 100 *μ*l of the cell suspension was mixed with a reporter plasmid mix using a 100 *μ*l Neon tip. For analysis of oxidative stress response, the antioxidant response reporter plasmid pNL[NlucP/ARE/Hygro] and reference reporter plasmid pGL4.13[luc2/SV40] were mixed at a ratio of 100 : 1 and a final concentration of 1 *μ*g/*μ*l. Electroporation was performed at 1475 volts with a pulse width of 20 ms and a pulse number of 2. The transfected cell suspension was diluted in 5 ml prewarmed medium (SMC-GM2) without antibiotics, and 100 *μ*l per well was seeded into white 96-well plates (resulting in 20000 cell/well). Cells were incubated at 37°C and 5% CO_2_ overnight to recover before further treatments were performed as indicated. After transfection and recovery, cells were treated with human AngII (Sigma, Merck, Darmstadt, Germany) in 80 *μ*l medium/well at different concentrations for 3 h or 24 h, respectively, or left untreated (control) as described in the figure legends. For detection of luciferase activity, dual luminescence was measured using the Nano-Glo Dual-Luciferase Reporter Assay System (Promega, Walldorf, Germany), following the protocol of the manufacturer. Briefly, plates were equilibrated to room temperature and 80 *μ*l of One-Glo Ex Luciferase reagent was added to each well. Plates were mixed for 3 min at 240 rpm, and firefly luminescence was measured using a TECAN spark multiplate reader. Next, NanoDLR Stop & Glo Reagent was prepared (diluted 1 : 100 in buffer) and 80 *μ*l was added to each well. Plates were mixed by shaking for 3 min and incubated at room temperature for 10 min. NanoLuc luciferase was measured, again using the TECAN spark reader. Relative luminescence (RLU) was calculated by dividing the signal intensities of the NanoLuc luciferase by the signal intensities of the firefly luciferase.

### 2.5. Detection and Quantification of Hydrogen Peroxide Levels Using ROS-Glo H_2_O_2_ Assay

VSMC were seeded at 1 × 10^4^ cells per well in a 96-well plate and serum-deprived overnight. Detection of H_2_O_2_ was performed with ROS-Glo H_2_O_2_ assay (Promega, Walldorf, Germany) as described by the manufacturer. Briefly, cells were treated for different lengths of time with 40 *μ*l complete medium (control) or AngII at 1 *μ*mol/l and/or MitoQ (mitoquinone mesylate, Biozol, Eching, Germany) at 100 nmol/l in 40 *μ*l complete SMC-GM2, at 37°C and 5% CO_2_ in a humidified atmosphere. For detection after 2 h, 10 *μ*l of H_2_O_2_ substrate solution was added immediately at the start of the treatment. For detection after 6 h and 24 h, 10 *μ*l of H_2_O_2_ substrate solution was added 6 h before measurement. After treatment, 50 *μ*l of ROS-Glo Detection Solution was added and cells were incubated for 20 min, before luminescence was measured with a TECAN plate reader.

### 2.6. Detection and Quantification of Superoxide Anion (O_2_^−^) Levels Using CellROX Green Assay

VSMCs were seeded at 1 × 10^4^ cells per well in a 96-well plate and serum-deprived overnight. Detection of O_2_^−^ was performed with CellROX Assay (Thermo Fisher, Invitrogen) as described by the manufacturer. Briefly, cells were treated with complete medium (control) or AngII at 1 *μ*mol/l and/or MitoQ at 100 nmol/l in complete SMC-GM2, as described in the figure legends. After treatment, cells were washed three times with phosphate-buffered saline (PBS) and 50 *μ*l of CellROX green reagent was added. Cells were incubated for 30 min at 37°C and 5% CO_2_ in a humidified atmosphere, before fluorescence was measured with a TECAN plate reader at an excitation wavelength of 485 nm and an emission wavelength of 535 nm.

### 2.7. NADP+/NADPH Assays

Analysis of total NADP+/NADPH levels was performed with NADP/NADPH-Glo Assay (Promega, Walldorf, Germany), according to the instructions of the manufacturer. Briefly, VSMCs were seeded in 96-well plates at different densities (1 × 10^3^, 4 × 10^3^, and 8 × 10^3^ cells/well) in 100 *μ*l complete growth medium and incubated at 37°C and 5% CO_2_ overnight. Next, 50 *μ*l of NADP/NADPH-Glo Detection Reagent (prepared as recommended by the manufacturer) was added to each well and luminescence was measured as relative light units (RLU) after 60 min with a TECAN Spark multiplate reader. For analysis of the NADPH/NADP+ ratio, 8000 cells per well were seeded with or without AngII (1 *μ*M) in 100 *μ*l complete growth medium and incubated overnight. Cells were washed with DPBS and lysed with 0.2 N NaOH solution containing 1% DTAB (dodecyltrimethylammoniumbromid) by shaking of the plates for 15 min. Cell lysates (100 *μ*l each) were used immediately for analysis or cryopreserved at -80°C, before oxidized (NADP+) and reduced (NADPH) levels were analysed separately, as recommended by the manufacturer. Briefly, 50 *μ*l of each lysate was transferred to an empty well for acid treatment (NADP+ measurement) and 50 *μ*l of each lysate was transferred to another well for base treatment (NADPH measurement). To measure NADP+, 25 *μ*l of 0.4 N HCl was added per well and heated to 60°C for 15 min. To measure NADPH, samples were directly heated to 60°C for 15 min. Samples were equilibrated for 10 min at room temperature, before 25 *μ*l of 0.5 M Trizma® base was added to each well of the acid-treated cells, and 50 *μ*l of HCl/Trizma® solution was added to each well of base-treated samples. Next, 100 *μ*l of NADP/NADPH-Glo™ Detection Reagent was transferred to each well and luminescence was measured after 60 min with a TECAN Spark multiplate reader.

### 2.8. Immunofluorescence Staining of *γ*-H2A.X and Analysis of DNA Damage

Cells were seeded on cover slips in complete SMC-GM2 per well and grown overnight at 37°C and 5% CO_2._ The medium was replaced with AngII-containing medium at different concentrations (0 nM, 10 nM, 100 nM, or 1000 nM) and incubated for another 24 h. H_2_O_2_- (100 *μ*M) containing medium was used as a positive control. Cells were washed three times with Dulbecco's phosphate-buffered saline (DPBS), with 0.1% Tween 20 and fixed in 4% paraformaldehyde for 10 min at room temperature. After additional washing, cells were permeabilized with 0.1% Triton X-100 in DPBS for 10 minutes and blocked with 2%albumin fraction + 2%FBS in washing buffer for 1 hour. Primary antibody (Phospho-Histone H2A.X (Ser139), Cell Signaling Technology Europe, Frankfurt, Germany, #2577; dilution 1 : 400) was added overnight in a humidified chamber at 4°C. Cover slips were washed again three times, before the secondary antibody (Anti-rabbit IgG Fab2 Alexa Fluor 488, Cell Signaling Technology Europe, Frankfurt, Germany, #4412; dilution 1 : 500) was added for one hour. After final washing for three times, ProLong Antifade Gold Mountant with DAPI (Thermo Fisher Scientific, Waltham, USA) was applied to counterstain cell nuclei. Cells were visualized under a fluorescence microscope (Zeiss Axiostar Plus) with 40x objective, and photos were taken for further analysis. Eight to 15 images were captured for each treatment group per cell line. For quantification, the total number and the number of *γ*-H2A.X-positive nuclei were determined from the pictures to obtain the percentage of positive nuclei.

### 2.9. Immunofluorescence Staining of p21Waf1/Cip1

Cells were seeded on cover slips and grown overnight at 37°C and 5% CO_2_ in SMC-GM2 with reduced serum (1% supplement) to synchronize the cell cycle. After three times washing with PBS, cells were fixed and permeabilized as described above, before blocking buffer (5% goat serum, 0.3% Triton™ X-100 in PBS) was added for 1 h. Primary antibody (p21Waf1/Cip1(12D1), Cell Signaling Technology Europe, Frankfurt, Germany, #2947; dilution 1 : 500) was added overnight in a humidified chamber. Further processing and addition of the secondary antibody were performed as described above. Cells were visualized under a fluorescence microscope (Zeiss Axiostar Plus) with 20x objective, and photos were taken for further analysis. Two to three images were captured for each treatment group per cell line. Quantification of p21-positive nuclei was performed with FIJI/ImageJ.

### 2.10. WST-1 Proliferation Assay

The WST-1 proliferation assay (Sigma Aldrich, Taufkirchen, Germany) was used for analysis, as described by the manufacturer. Briefly, cells were grown overnight in 96-well plates at a density of 1 × 10^4^ cells/well in 100 *μ*l SMC-GM2 with reduced serum (1% supplement) to synchronize the cell cycle. Assays were initiated by changing the medium to complete SMC-GM2, and cells were grown for another 0 h (start), 24 h, 48 h, and 72 h as described in the figure legends. For analysis, 10 *μ*l of WST-1 proliferation reagent was added to each well and absorbance was measured at 450 nm versus 620 nm with a TECAN Spark reader, as recommended by the manufacturer. To determine the relative proliferation rate, all data (A450-A620) were normalized to the absorbance at the start. All experiments were performed in triplicate.

### 2.11. Apoptosis Assay

Apoptotic cells were quantified by using the bioluminescent Caspase-Glo 3/7 Assay Systems (Promega, Walldorf, Germany) as described by the manufacturer. Briefly, cells were grown overnight in 96-well plates at a density of 1 × 10^4^ cells/well in SMC-GM2 with reduced serum (1% supplement) to synchronize the cell cycle. Assays were initiated by changing the medium to 50 *μ*l complete SMC-GM2, and cells were grown for another 0 h (start), 24 h, 48 h, and 72 h as described in the figure legends. For analysis, 50 *μ*l of the Caspase-Glo® 3/7 Reagent was added to each well and Caspase-Glo 3/7 activity was measured as relative light units (RLU) with a TECAN Spark reader.

### 2.12. Statistical Analysis

Three to eight individual cell cultures were analysed in triplicate to quintuplicate for each experiment. Data were processed by using the GraphPad Prism software (version 9.0.0). Pairwise comparison was performed using the Mann–Whitney *U* test or Wilcoxon rank sum test as nonparametric tests or Student's *t*-test as parametric test. Multiple comparisons were performed by 2-way ANOVA and additional tests as described in the figure legends.

## 3. Results

### 3.1. Morphological Analysis Indicates Increased Mitochondrial Fission in AAA-SMC

**Figure 1:**
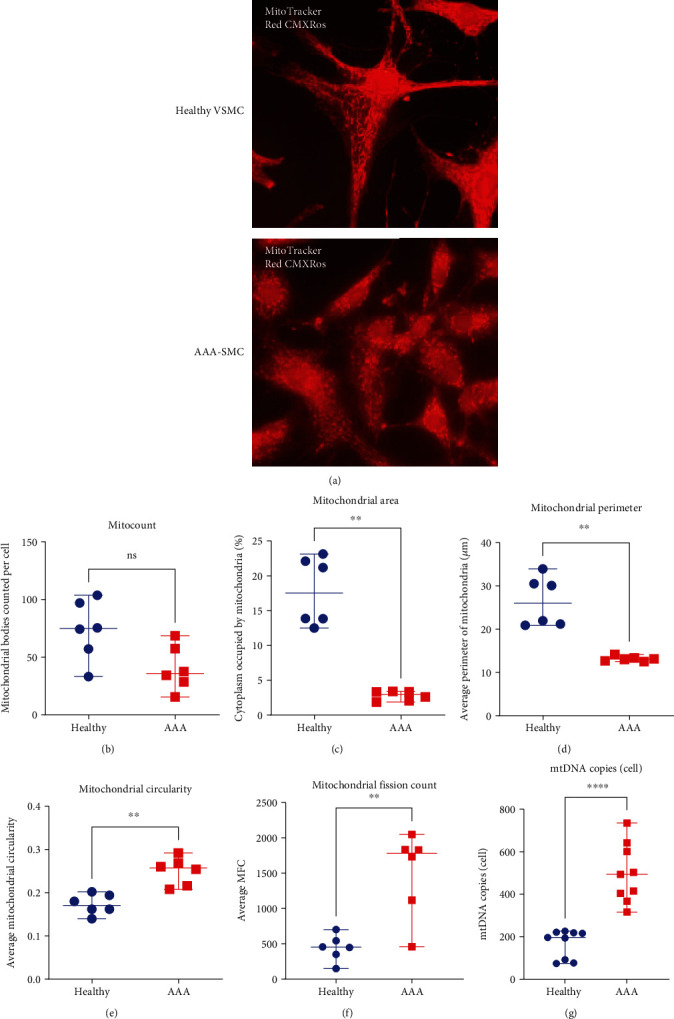
(a) Representative images of healthy VSMC (*n* = 6 cultures) and AAA-SMC (*n* = 6 cultures) stained with MitoTracker Red CMXRos. Mitochondrial particle count per cell (b), mitochondrial area (c), mitochondrial perimeter (d), and mitochondrial circularity (e) were determined by ImageJ as described in Materials and Methods. (f) Mitochondrial fission count was calculated using ImageJ data (mitochondria count × 100/mitochondrial area (%area of cell) per cell = MFC). (g) The number of mitochondrial DNA copies per cell was determined by real-time PCR. Data points represent the means of each culture (4-6 cells were captured per cell culture) and are shown with median and range. Data were analysed with the Mann–Whitney *U* test; ^∗∗^*p* < 0.01 and ^∗∗∗∗^*p* < 0.0001, ns: not significant.

To investigate mitochondrial morphology and dynamics, AAA-SMC and healthy VSMC were stained with MitoTracker Red CMXRos (Figure 1(a)) and mitochondria were analysed microscopically for four parameters as previously described [21]. The mitochondrial particle count per cell did not differ significantly between AAA-SMC and healthy VSMC (Figure 1(b)). In contrast, the percentage of cytoplasm occupied by mitochondria was significantly reduced in AAA-SMC (median = 3.0%) compared with healthy VSMC (median = 17.5%), indicating a lower mitochondrial mass in AAA-SMC (Figure 1(c)). The median mitochondrial perimeter was as well significantly lower in AAA-SMC than in healthy VSMC (median_AAA-SMC_: 13.0 *μ*m versus median_healthy VSMC_: 26.0 *μ*m) (Figure 1(d)).

Finally, the average circularity of mitochondria was significantly higher in AAA-SMC compared with healthy VSMC (Figure 1(e)). Taken together, AAA-SMC contained less mitochondrial bodies, which were smaller and rounder than those in healthy VSMC. Accordingly, the mitochondrial fission count was elevated in AAA-SMC (Figure 1(f)), indicating increased mitochondrial damage in these cells.

Because of the different degree of fusion of mitochondria, the number of mitochondrial bodies does not exactly reflect their individual number. We therefore performed a mitochondrial DNA- (mtDNA-) specific real-time quantitative PCR [18] using the human MTND1 gene, which is encoded in the mitochondrial genome, to generate a standard curve. The mtDNA copy number per cell was significantly higher in AAA-SMC (median = 494 copies/cell) than in healthy VSMC (median = 197 copies/cell, Figure 1(g)).

### 3.2. The Antioxidant Stress Response Is Affected in AAA-SMC

Given that mitochondrial dysfunction is particularly driven by oxidative stress [23, 24], we next determined the antioxidant stress response of AAA-SMC versus healthy VSMC. Cells were transfected with a plasmid containing the antioxidant response element (ARE) driving NanoLuc luciferase as a reporter gene. ARE is an enhancer element that is found in several genes encoding detoxification enzymes and initiates a pathway to protect cells from oxidative stress-induced cell death when activated by the transcription factor Nrf2 (nuclear factor erythroid 2-related factor 2) [25, 26].

**Figure 2:**
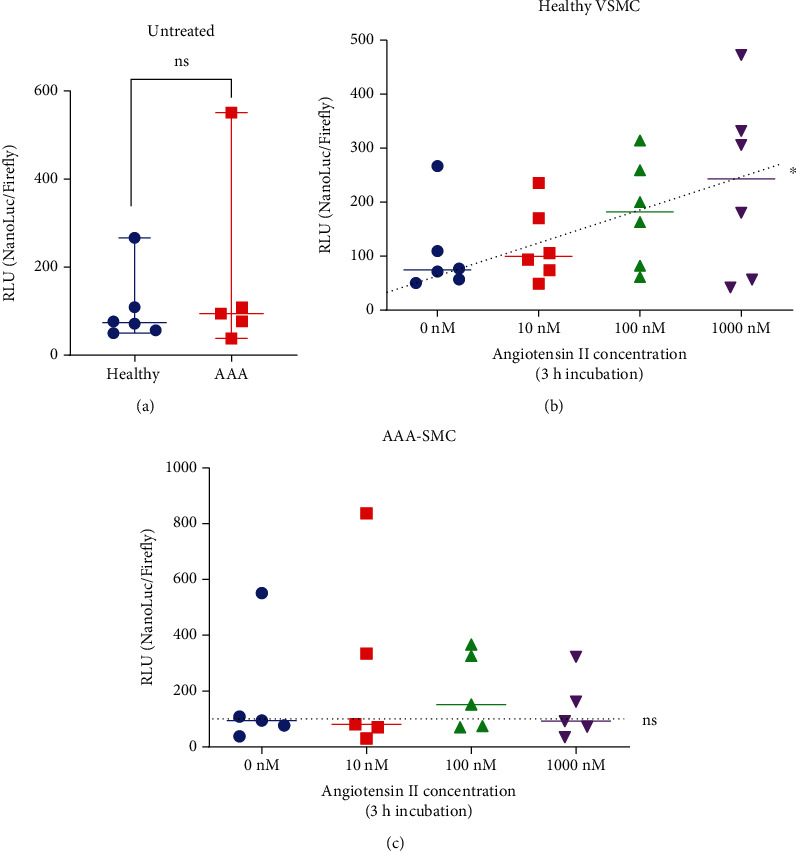
Antioxidative response element (ARE) activity was analysed in healthy (*n* = 6) and AAA-VSMC (*n* = 5) cultures following 3 h of AngII treatment at 0, 10, 100, and 1000 nM concentrations. ARE activity was determined as relative luminescence (RLU) of the ARE-driven NanoLuc luciferase divided by the RLU of a reference firefly luciferase. (a) ARE activity in untreated cells. Data points represent the means of four replicates per culture and are shown with median and range of all cultures. Statistical analysis was performed with the Mann–Whitney *U* test. ns: not significant. (b, c) ARE activity in healthy VSMC (b) and AAA-SMC (c) as a function of 3 h treatment with different doses of AngII. Data points represent the means of four replicates per culture and are shown with median. Ordinary one-way ANOVA was performed to test for a linear trend. ^∗^*p* < 0.05. The dotted line represents the trend line.

In untreated cells, ARE activity did not differ between healthy VSMC and AAA-SMC (Figure 2(a)). To increase oxidative stress, we challenged the cells with AngII, which has been reported to induce ROS production, thereby mediating oxidative DNA damage and senescence in VSMC [27, 28]. Treatment of the cells with increasing doses of AngII for 3 h resulted in a significant linear increase of the ARE response in healthy VSMC (Figure 2(b)) but not in AAA-SMC (Figure 2(c)). No difference was observed after 24 h of AngII treatment (supplementary figure S1). This suggests that the response to AngII-mediated oxidative stress is affected in AAA-SMC. Results of the individual cell cultures may be found in supplementary figure S2.

### 3.3. Oxidative Stress and ROS Production

**Figure 3:**
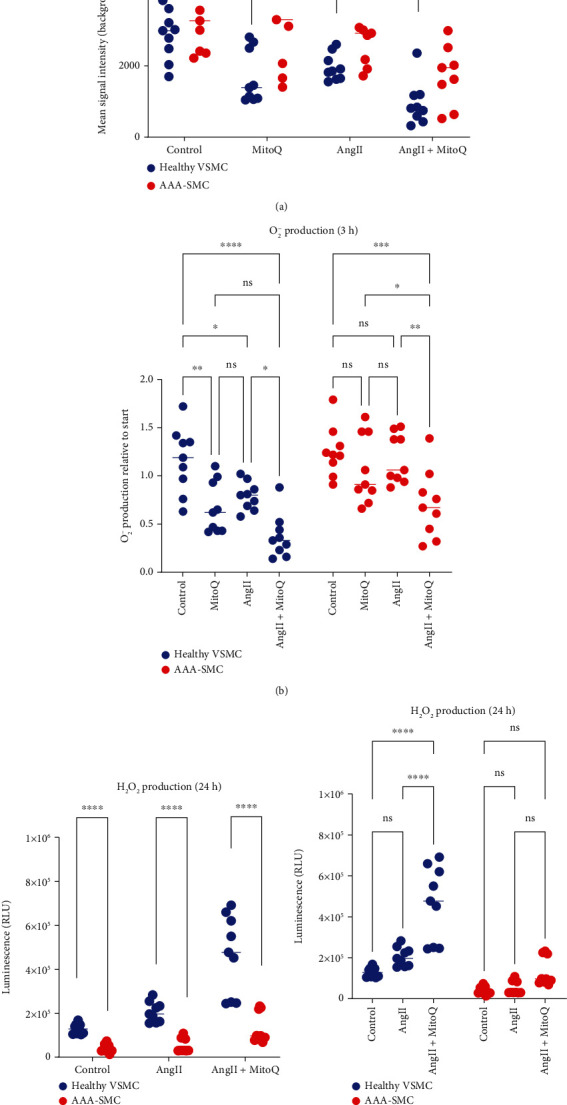
ROS production in healthy VSMC and AAA-SMC. (a, b) Analysis of O_2_^−^ production in controls and after 3 h of treatment with 100 nM MitoQ, or 1 *μ*M AngII, or a combination of both. Cells were stained with CellROX green reagent, and fluorescence was measured with a TECAN multiplate reader. Data are shown as mean signal intensity after background subtraction (a) or as absorbance relative to start (b) of three healthy and three AAA-SMC cultures analysed in triplicate. (c, d) Analysis of H_2_O_2_ amounts in controls and after 24 h of treatment with 1 *μ*M AngII alone or in combination with 100 nM MitoQ. Detection of H_2_O_2_ was performed with a luminescent ROS-Glo H_2_O_2_ assay. Data are shown as relative light units (RLU) of three healthy and three AAA-SMC cultures analysed in triplicate and were analysed by multiple Mann–Whitney tests (a, c) or by 2-way ANOVA and Šidák's multiple comparison test as a post hoc test (b, d). ns: not significant; ^∗^*p* < 0, 05, ^∗∗^*p* < 0.01, ^∗∗∗^*p* < 0.001, and ^∗∗∗∗^*p* < 0.0001.

Increased oxidative stress has been found in human and mouse AAA tissues [10, 13, 29, 30] and was mainly localized to infiltrating macrophages within the adventitia [13], infiltrating inflammatory cells, and VSMC [30], by using in situ imaging. To address mitochondrial ROS production in VSMC *in vitro*, we next determined the levels of superoxide anions (O_2_^−^) and hydrogen peroxide (H_2_O_2_) in untreated and AngII-challenged SMC cultures in the presence or absence of the mitochondrial-specific antioxidant MitoQ, which was shown to reduce ROS production in VSMC [28]. After three hours of treatment, the amount of O_2_^−^ was significantly higher in AAA-SMC than in healthy VSMC (Figure 3(a)), and MitoQ treatment significantly decreased the amount of O_2_^−^ in healthy VSMC, both in controls and in AngII-challenged cell cultures (Figure 3(b)).

In AAA-SMC, the amount of O_2_^−^ was also reduced by MitoQ, but to a lower extent. This indicates that O_2_^−^ production derived from mitochondria is increased in AAA-SMC. No significant differences in the amount of O_2_^−^ were observed after 24 h of treatment (supplementary figure S3a and b). In contrast, the amount of H_2_O_2_ was unaffected after two hours of treatment (supplementary figure S3c), both in healthy and in AAA-SMC but displayed clear differences after 24 h of treatment, when H_2_O_2_ levels were clearly higher in healthy VSMC cultures than in AAA-SMC (Figure 3(c)). Moreover, the amount of H_2_O_2_ was significantly increased in MitoQ-treated cells after 24 h, again with a stronger effect in healthy VSMC than in AAA-SMC (Figure 3(d)).

AngII treatment elicited similar effects on ROS production as MitoQ treatment. After 3 h of treatment, O_2_^−^ production was significantly reduced in healthy VSMC (Figure 3(b)), resulting in an increased H_2_O_2_ production after 24 h (Figure 3(d)). Again, the effect of AngII on ROS production was much weaker and not significant in AAA-SMC (Figures 3(a)–3(d)).

### 3.4. AAA-SMCs Contain Less NADPH/NADP+ Than Healthy VSMC

**Figure 4:**
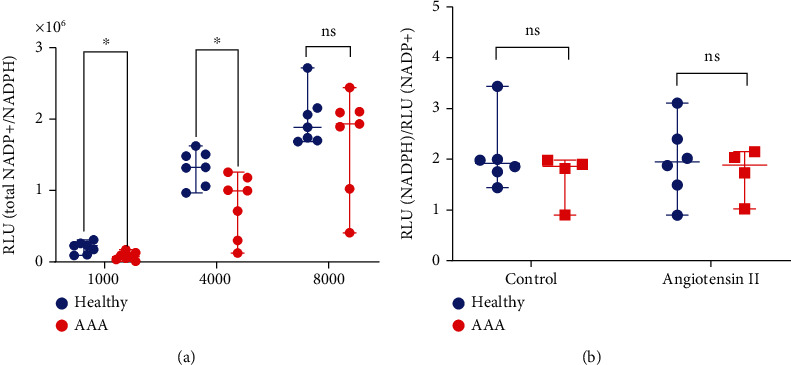
NADP+/NADPH metabolism. (a) Total NADP+/NADPH levels in untreated healthy VSMC (*n* = 7) and AAA-SMC (*n* = 7) cultures for varying cell densities. (b) NADPH to NADP+ ratio in healthy VSMC (*n* = 6) and AAA-SMC (*n* = 4) lines, left untreated or after treatment with 1 *μ*M AngII for 24 hours. All measurements were performed in triplicate. Data are shown as median with range and were analysed pairwise with the Mann–Whitney *U* test, ^∗^*p* < 0, 05; ns: not significant.

Nicotinamide adenine dinucleotide phosphate (NADP+) and its reduced form (NADPH) play critical roles in both antioxidation system and generation of oxidative stress [31, 32]. Because they are predominantly located in mitochondria [31], we used a bioluminescent NADP/NADPH-Glo Assay to determine the total amount and the ratio of NADPH/NADP+ as surrogates for mitochondrial function in VSMC. The median total amount of NADP+/NADPH was found to be significantly lower in AAA-SMC than in healthy VSMC (Figure 4(a)), indicating a reduced metabolic activity. Treatment of the cells with AngII did not change the total amount of NADPH/NADP+, neither in healthy nor in AAA-SMC (supplementary figure S4). In contrast to the total amount, the NADPH/NADP+ ratio did not differ significantly between healthy and AAA-SMCs (Figure 4(b)).

### 3.5. Elevated *γ*-H2A.X Presence Indicates Increased DNA Damage in AAA-SMC in Response to AngII

**Figure 5:**
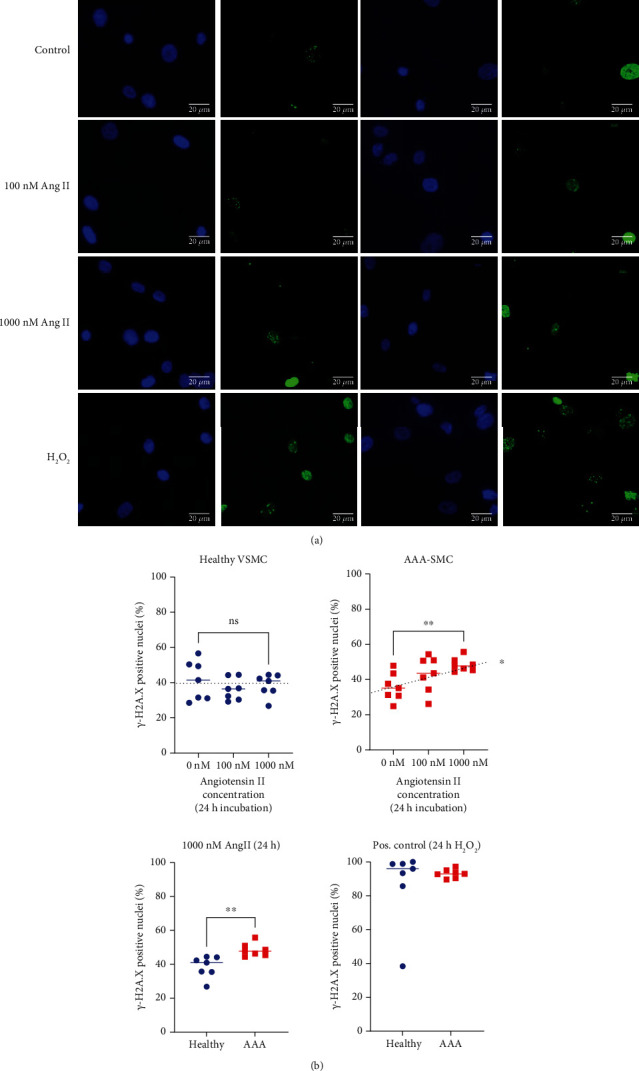
DNA damage in response to AngII in AAA-SMC (*n* = 7 cultures) versus healthy VSMC (*n* = 7 cultures). Cells were incubated with increasing concentrations of AngII for 24 h, respectively; fixed; and immunostained with an anti-*γ*-H2A.X antibody as described in Materials and Methods. 100 *μ*M H_2_O_2_ was used as a positive control for maximal DNA damage after 24 h. (a) Representative images of healthy VSMC and AAA-SMC after treatment with AngII for 24 h. Scale bars = 20 *μ*m. (b) Graphical representation of the image evaluation. Data points represent the mean percentage of *γ*-H2A.X-positive nuclei per total nuclei for each cell culture and are presented with median of *n* = 7 AAA-SMC cultures and *n* = 7 healthy VSMC cultures. Eight to fifteen images of each culture were evaluated for analysis. Statistical analysis was performed pairwise with the Mann–Whitney *U* test, ^∗∗^*p* < 0.01. Ordinary one-way ANOVA was performed to test for a linear trend. ^∗∗^*p* < 0.01; ns: not significant.

Excessive ROS levels produced by dysfunctional mitochondria can cause severe oxidative damage to macromolecules, especially the DNA [33]. We therefore evaluated DNA double-strand breaks in AAA-SMC (*n* = 7 cultures) and healthy VSMC (*n* = 7 cultures) by immunostaining for *γ*-H2A.X foci after 3 h (Supplementary Figure S5) and 24 h (Figure 5) of treatment with different concentrations of AngII. Gamma-H2A.X is a phosphorylated histone protein, which is required for checkpoint-mediated cell cycle arrest and DNA repair following double-strand breaks and represents a very early event in DNA damage response [34].

Gamma-H2A.X positive nuclei were observed to appear in a wide spectrum from singular small foci to staining of the whole nucleus (Figure 5(a)). The percentage of *γ*-H2A.X-positive nuclei did not differ significantly between AAA-SMC and healthy VSMC in untreated cells and in response to short-time AngII exposures (Supplementary Figure S5). When the cells were treated with 1 *μ*M AngII for 24 h, the percentage of *γ*-H2A.X-positive nuclei increased significantly in AAA-SMC, when compared with untreated cells and with healthy VSMC, indicating an increased number of cells with double-strand breaks. (Figure 5(b)).

### 3.6. AAA-SMCs Are Subject to Earlier Growth Arrest Than Healthy VSMC

**Figure 6:**
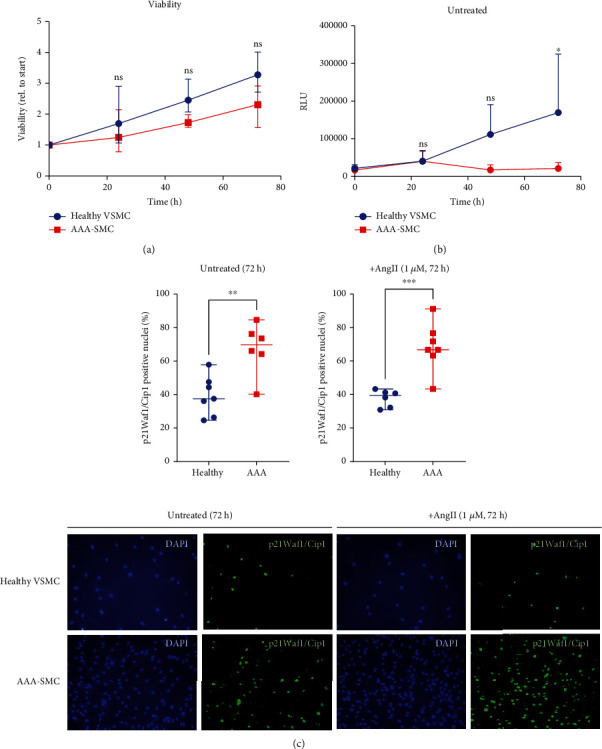
AAA-SMCs are subject to earlier growth arrest than healthy VSMCs. Cells were grown with serum withdrawal overnight before full medium was added with or without different concentrations of AngII. (a) Viability at different time points of untreated healthy VSMC and AAA-SMC as determined by WST-1 proliferation assay. Data show the means and ranges of *n* = 3 healthy VSMC and *n* = 3 AAA-SMC cultures analysed in triplicate. ns: not significant. (b) Apoptosis at different time points of untreated healthy VSMC and AAA-SMC as determined by Caspase-Glo 3/7 Assay. Data show the means and ranges of *n* = 3 healthy VSMC and *n* = 3 AAA-SMC cultures that were analysed in triplicate. Data were statistically analysed with 2-way ANOVA and Šídák's multiple comparison test as a post hoc test using the GraphPad Prism software. ^∗^*p* < 0.05; ns: not significant. (c) Upper panel: percentage of p21Waf1/Cip1-positive nuclei in untreated and AngII-treated cell cultures. Data represent the median and range of *n* = 3 healthy VSMC and *n* = 4 AAA-SMC cultures, of which two or three images were taken for analysis. Data were statistically analysed with unpaired *t*-tests using the GraphPad Prism software. ^∗^*p* < 0.05, ^∗∗^*p* < 0.01, and ^∗∗∗^*p* < 0.001. (c) Lower panel: representative images of samples grown for 72 hours without or with (1 *μ*M) AngII and fluorescently labelled with p21Waf1/Cip1.

The identification of mitochondrial dysfunction, altered metabolic activity, and increased sensitivity towards DNA damage prompted us to investigate proliferation of AAA-SMC versus healthy VSMC by using a WST-1 proliferation assay and a Caspase-3/7 Apoptosis Assay. The mean number of viable cells was similar in healthy VSMC and AAA-SMC at 24 h, 48 h, and 72 h after initiation of the assay (Figure 6(a) and Supplementary figures 6 and 7). In contrast, the proportion of apoptotic cells in healthy VSMC increased continuously, whereas it remained unchanged in AAA-SMC (Figure 6(b)).

Thus, healthy VSMCs balance apoptosis by increased proliferation, whereas AAA-SMCs grow slowly, despite very little apoptosis. Together, this indicates that AAA-SMCs stop proliferating earlier than healthy VSMCs. In line with this observation, the amount of the cell cycle inhibitor and DNA damage checkpoint marker p21Waf1/Cip1 was increased in AAA-SMC grown for 72 hours (Figure 6(c)), indicating that the cells do not pass the DNA damage/G2 checkpoint. No additional effect on cell viability and p21 expression was observed, when the cells were treated with AngII for 72 hours (Figure 6(c) and supplementary figures 6 and 7), indicating that AngII had no effect on the proliferation rate.

## 4. Discussion

In this paper, we characterized mitochondrial oxidative stress in AAA-SMC in comparison to healthy VSMC, grown *in vitro*. Our data present evidence that mitochondria in AAA-derived VSMC are permanently and more severely damaged than those of healthy VSMC derived from age-matched controls. We have also highlighted the role of AngII, which appears to exert different effects on ROS production in healthy and aneurysm-damaged cells, respectively. So far, most studies of VSMC physiology, metabolism, and therapeutic targets have been performed in single cultures of healthy cells, ignoring the fact that many processes may be different in cells from diseased tissue and between individuals. Therefore, our approach was to separately study and compare the mitochondrial function of individual VSMC cultures from different healthy and aneurysmal aortas, at early passages after isolation from the tissue.

Under physiological conditions, ROS production in vascular mitochondria is at a low level. However, overproduction of these ROS can trigger pathophysiological reactions and thus impair vascular function [35]. This is because, despite antioxidant repair mechanisms, excessive amounts of ROS can eventually no longer be compensated and generate oxidative stress. Over many years, mitochondria can counteract this stress by regenerating permanently. A cycle of biogenesis, fusion, and fission guarantees the preservation of function and the disposal of damaged mitochondria by mitophagy [36]. However, with increasing age, dysfunctional mitochondria accumulate and thus also increase oxidative stress [37].

Here, we used *in vitro* cultures of AAA-derived and age-matched healthy VSMC to decipher the impact of mitochondrial oxidative stress in connection with AAA. We observed that mitochondrial damage in AAA-SMC was permanent and the resulting oxidative stress could not be compensated for as well as in healthy VSMC. Indeed, AAA-SMC contained less mitochondrial bodies, which were smaller and rounder than those in healthy VSMC. In addition, mitochondrial fission was significantly increased. Our findings are in line with previous reports. Increased fission of mitochondria has been described as a therapeutic target of AAA in humans and rodent abdominal aortic VSMC [38]. The authors speculated that the underlying mechanism involves upregulation of Drp1-mediated mitochondrial fission which results in a proinflammatory phenotype. In addition, exposure to AngII, one of the main inducers of AAA in mouse models, has been shown to induce mitochondrial fission in cultured rat aortic VSMC [39]. In general, mitochondrial fission creates smaller, more discrete mitochondria, which are more capable of generating ROS, and a higher fission-to-fusion rate has been associated with many diseases including cancer and cardiovascular diseases [40]. Under physiological conditions, fission is used to distribute mitochondria evenly among daughter cells during cell division. In addition, defective mitochondria can be separated and disposed of in this way before they cause damage or induce cell death [36]. Accordingly, our findings of increased fission in AAA-SMC may be interpreted as a response to the accumulation of damaged mitochondria that occurred during the course of aortic degeneration.

The higher number of mtDNA copies in AAA-SMC detected here was unexpected. Filamentous, elongated mitochondria contain several (6-8) mtDNA copies per mitochondrial nucleoid, whereas small mitochondria contain little to no mtDNA [41]. Thus, the higher mtDNA copy number seems to be at odds with the smaller and rounder mitochondria that were detected in AAA-SMC. However, reduction of mitochondrial particles after fission also requires functional mitophagy. Moreover, mtDNA can be released from defective mitochondria and accumulate outside of mitochondria if not properly removed. Thus, the high mtDNA copy number in combination with an increased fission to fusion rate may indicate defects in mitophagy in AAA-SMC, as it has been suggested by others [42]. Clear evidence of defective mitophagy in VSMC and its underlying causes in the course of AAA disease is still pending.

Another important observation described in this paper is the role of AngII, which appears to exert different effects on healthy and aneurysm-derived cells, respectively. A clear dose-dependent response to AngII via the antioxidative stress element ARE was only observed in healthy VSMC but not in AAA-SMC. AngII has been repeatedly described as a significant stimulus for ROS production in the vasculature and a trigger of senescence in aortic VSMC [28, 43–45]. All of these reports have used healthy VSMC for their analyses. Our data are in line with these reports, demonstrating that ARE response increased with AngII in healthy VSMC cultures 3 h after AngII treatment. However, to our knowledge, this is the first report that presents evidence for an alteration in the antioxidative stress response via the ARE pathway in AAA-SMC. Activation of the ARE element in genes encoding detoxification enzymes by the transcription factor Nrf2 protects cells from oxidative stress-induced cell death. The Nrf2/ARE-pathway has been shown to be important for protection against H_2_O_2_-induced inflammation and oxidative stress in cardiomyocytes [46], and alterations in this cascade have been reported to be associated with neurodegenerative diseases [47]. Given the importance of ROS detoxification, it will be very interesting to investigate Nrf2 and other components of this pathway for their expression and function in vascular cells in connection with AAA. It is possible that VSMC respond differently to AngII depending on the activity of this signalling pathway. This might explain in part the heterogenous stress response observed in AAA-SMC and has important implications for future use of pharmaceutical therapeutics.

As a result of reduced ARE activation and thus expression of detoxification enzymes, an accumulation of ROS would be expected. Indeed, our data demonstrated increased O^2-^ levels in AAA-SMC. The fact that mitochondria-targeted MitoQ was able to reduce O^2-^ levels in both controls and AngII-challenged healthy VSMCs, but not in AAA-SMCs, suggests involvement of mitochondria in activation of the Nrf2/ARE cascade, as it was demonstrated in cardiomyocytes [46]. Interestingly, the level of another ROS, H_2_O_2_, was clearly higher in healthy VSMC cultures than in AAA-SMC after 24 h. Moreover, the amount of H_2_O_2_ was significantly increased in MitoQ-treated cells after 24 h, again with a stronger effect in healthy VSMC than in AAA-SMC. Overall, this indicates that H_2_O_2_ production follows mitochondrial O_2_^−^ production and is reduced in AAA-SMC. Considering that mitochondrial antioxidant defence is largely performed by manganese superoxide dismutase (MnSOD), which converts O_2_^−^ to H_2_O_2_ and was shown to play an important role in vascular function [48], our data suggest MnSOD as another target that might be affected in AAA-SMC. MnSOD is considered the chief ROS scavenging enzyme in the cell, and reduced expression and/or activity of MnSOD result in diminished antioxidant capacity of the cell, including alterations of metabolic function. The resulting excess of O_2_^−^ radicals contributes to the production of other ROS that further damage mitochondria [49]. Interestingly, a recent study demonstrated that mitochondrial-targeted therapies require mitophagy to prevent oxidative stress induced by MnSOD inactivation in cardiomyocytes [50]. Together with the alterations found here in mitochondrial dynamics and the reduced response to the antioxidant MitoQ, this supports the hypothesis of MnSOD inactivation in AAA-SMC.

The capability to balance NADPH and NADP+ was unaffected in both healthy VSMC and AAA-SMC, suggesting that the activity of NADPH oxidase which has previously been demonstrated to increase ROS production in VSMC [29] is not implied in oxidative stress generation, here. In contrast, the lower total amount of NADPH/NADP+ argues for a reduced mitochondrial mass in AAA-SMC, which is in line with our observations on mitochondrial dynamics.

Finally, the DNA in AAA-SMC appears to be more vulnerable towards high doses of AngII than that in healthy VSMC. Considering the differences in ARE activity, described above, this might be a consequence of their lack of an effective antioxidative stress response. The precise mechanism of this inadequate antioxidative stress response however remains to be elucidated.

Overall, we identified several distinct targets that may be impaired in AAA-SMC, thereby contributing to the development and persistence of oxidative stress in AAA: mitochondrial fission, mitophagy, the Nrf2/ARE pathway, and MnSOD. It is also possible that different alterations in these targets are present in AAA-SMC from different individuals, all leading to the same outcome, namely, a cascade of oxidative stress, inflammation, and degradation of the vessel wall. Future investigation will elucidate the roles of these targets and help to identify subsets of AAA patients that might benefit from different target-specific therapeutics to slow down the growth and prevent rupture of AAA.

A limitation of our study may be the origin of the healthy VSMCs that were used for control. While the AAA-SMCs were taken from the abdominal region of the aorta, the healthy VSMCs were exclusively from thoracic regions of the aorta, because healthy abdominal VSMCs are not commercially available. In fact, thoracic and abdominal VSMCs stem from different embryonic lineages and there is evidence for different vulnerability and different responses to stress factors between thoracic and abdominal VSMCs [51–53]. Therefore, we cannot exclude that some of the differences in mitochondrial function and oxidative stress responses observed here are determined by their embryonic origin instead of reflecting damage associated with AAA progression. However, preliminary experiments with VSMC isolated from thoracic aneurysms and dissection argue for a disease-specific stress response rather than an embryologically determined one. Using the same experiments for investigation as described here, our studies showed that VSMCs from thoracic aneurysm and dissection respond more like VSMC from abdominal aneurysms than like healthy thoracic VSMCs (data not shown). A more detailed investigation of these differences is planned for future studies.

## 5. Conclusions

Based on our investigations on multiple cell cultures derived from healthy aorta and aortic aneurysms, we conclude that AAA-SMCs are persistently damaged. Our data strongly support the hypothesis that mitochondrial metabolism-generated free radicals, particularly O_2_^−^, account for mitochondrial dysfunction in AAA-SMC resulting in DNA damage and earlier senescence than healthy VSMC of age-matched adults. In addition, our data indicate a greater heterogeneity within AAA-SMC samples than within healthy VSMC which reflects the different responses to stress factors induced during aneurysm growth. Finally and in line with the observed heterogeneity, our data question the role of AngII in ROS-induced mitochondrial damage of AAA-SMC. AngII may drive ROS production in VSMC at early stages but seems to have little impact on advanced AAA-SMC. We conclude from our data that mitochondria in AAA-SMC may represent a worthwhile target for therapeutic intervention, for example, with mitochondria-targeted antioxidants to slow the growth of abdominal aortic aneurysms. However, these therapies may only be effective in some subgroups of AAA patients because of heterogeneous cellular responses, necessitating further studies.

## Data Availability

All the data supporting the conclusion were shown in the paper and in the supplementary files. Details of the primary data that support the findings of this study are available from the corresponding author upon reasonable request.

## Supplementary Materials

Supplementary Materials Supplementary table S1: characteristics of VSMC cultures. Supplementary Figure S1: ARE activity (relative to control, 0 nM) after 3 h and 24 h AngII treatment. Supplementary Figure S2: ARE activity in individual healthy VSMC and AAA-SMC cultures after treatment for 3 and 24 h with 0 nM, 10 nM, 100 nM and 1000 nM AngII. Supplementary figure S3: ROS production in healthy VSMC and AAA-SMC. (a and b) Analysis of O_2_^−^ production in controls and after 24 h of treatment with 100 nM MitoQ, or 1 *μ*M AngII, or a combination of both. Supplementary figure S4: total NADPH/NADP+ levels in response to AngII. Supplementary figure S5: DNA damage in response to AngII in AAA-SMC (*n* = 7 cultures) versus healthy VSMC (*n* = 8 cultures). Supplementary figure S6: viability of individual healthy VSMC cultures in response to AngII. Supplementary figure S7: viability of individual AAA-SMC cultures in response to AngII.
