# Supplementary material for: Mitochondrial Dysfunction and Increased DNA Damage in Vascular Smooth Muscle Cells of Abdominal Aortic Aneurysm (AAA-SMC)
Source: Oxid Med Cell Longev. 2023 Jan 25;2023:6237960. doi: 10.1155/2023/6237960 (PMC9891816; doi:10.1155/2023/6237960)
Supplement: Supplementary Materials — Supplementary table S1: characteristics of VSMC cultures. Supplementary Figure S1: ARE activity (relative to control, 0 nM) after 3 h and 24 h AngII treatment. Supplementary Figure S2: ARE activity in individual healthy VSMC and AAA-SMC cultures after treatment for 3 and 24 h with 0 nM, 10 nM, 100 nM and 1000 nM AngII. Supplementary figure S3: ROS production in healthy VSMC and AAA-SMC. (a and b) Analysis of O2− production in controls and after 24 h of treatment with 100 nM MitoQ, or 1 μM AngII, or a combination of both. Supplementary figure S4: total NADPH/NADP+ levels in response to AngII. Supplementary figure S5: DNA damage in response to AngII in AAA-SMC (n = 7 cultures) versus healthy VSMC (n = 8 cultures). Supplementary figure S6: viability of individual healthy VSMC cultures in response to AngII. Supplementary figure S7: viability of individual AAA-SMC cultures in response to AngII. [file 6237960.f1.pdf]

Supplementary materials to

Journal: Oxidative Medicine and Cellular Longevity

Title: Mitochondrial dysfunction and increased DNA damage in vascular smooth muscle cells of abdominal aortic aneurysm (AAA-SMC)

Authors: Bengi S. Tavris [1], Andreas S. Peters[1], Dittmar Böckler [1] and Susanne Dihlmann [1,*]

[1]Klinik für Gefäßchirurgie und endovaskuläre Chirurgie, Universitätsklinik Heidelberg, Im Neuenheimer Feld 420, 69120 Heidelberg
Correspondence should be addressed to Susanne Dihlmann: susanne.dihlmann@med.uni-heidelberg.de

Supplementary table S1: Characteristics of VSMC cultures

No	ID	origin	Age of patient (at extraction)	Sex
1	HAoSMC-424Z011	Healthy Aorta	14	male
2	HAoSMC-451Z003	Healthy Aorta	67	male
3	HAoSMC-452Z011	Healthy Aorta	68	male
4	HAoSMC-453Z011	Healthy Aorta	13	male
5	HAoSMC-470Z011	Healthy Aorta	51	male
6	HCSMC-1	Healthy carotid artery (carotid subclavian bypass)	67	male
7	HCSMC-2	Healthy carotid artery (carotid subclavian bypass)	82	male
8	AAA-SMC-1	AAA	65	male
9	AAA-SMC-2	AAA	49	male
10	AAA-SMC-3	AAA	69	male
11	AAA-SMC-4	AAA	58	male
12	AAA-SMC-5	AAA	74	male
13	AAA-SMC-6	AAA	59	male
14	AAA-SMC-7	AAA	66	male
15	AAA-SMC-8	AAA	65	male
16	AAA-SMC-9	AAA	73	male
17	AAA-SMC-10	AAA	67	male

Supplementary Figure S1: Antioxidative response element (ARE) activity in healthy and AAA-VSMC cultures

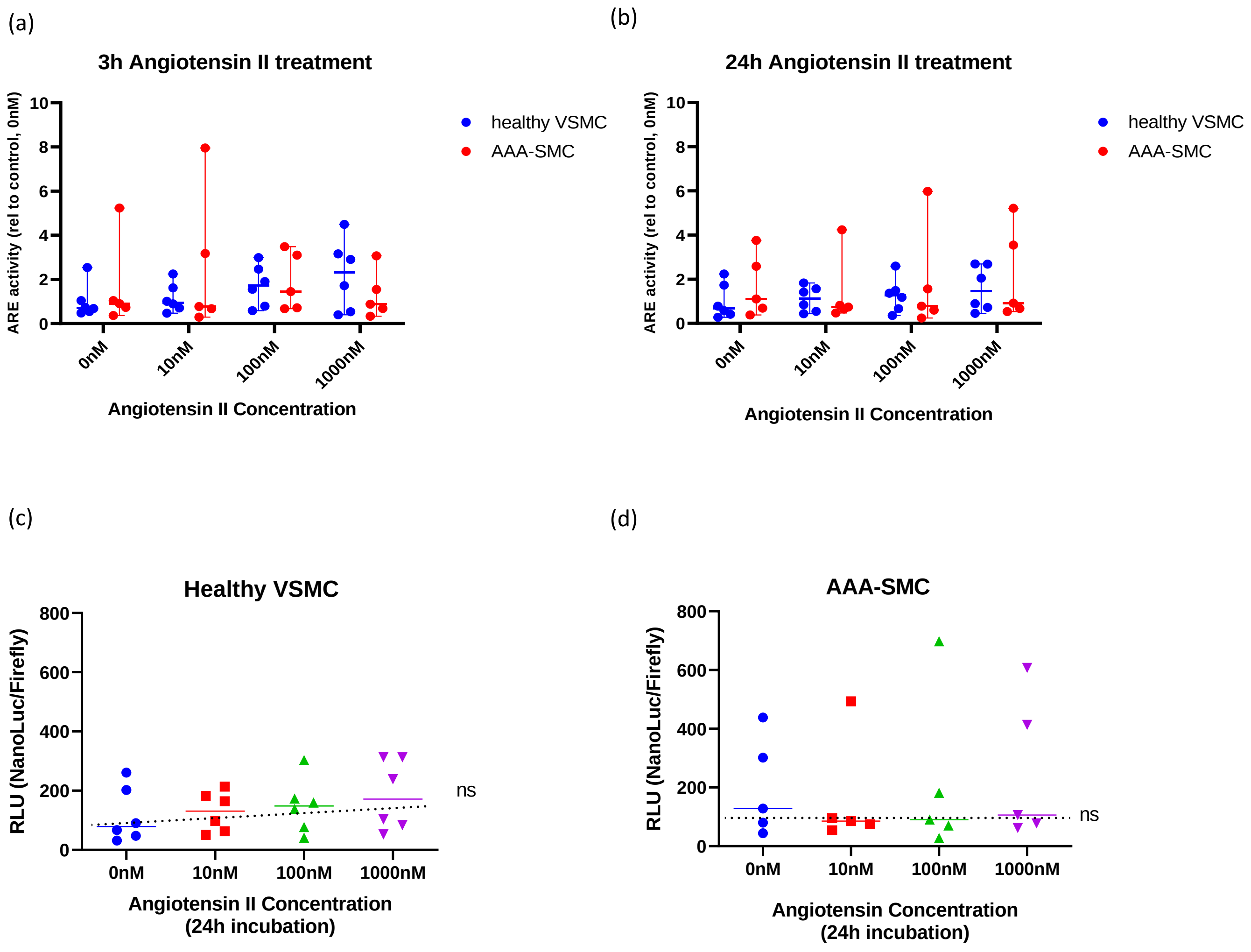

Supplementary Figure S1: Antioxidative response element (ARE) activity in healthy (n = 6) and AAA-VSMC (n = 5) cultures. ARE activity was determined as relative luminescence (RLU) of the ARE driven Nano-luc luciferase divided by the RLU of a reference firefly luciferase. Data points represent the mean RLU of four replicates per culture and are shown with median and range of all cultures.
(a) Comparison of the ARE activity between healthy VSMC and AAA-SMC after 3h of AngII treatment at different concentrations. (b) Comparison of the ARE activity between healthy VSMC and AAA-SMC after 24h of AngII treatment at different concentrations. (a and b) Statistical analysis was performed with Mann-Whitney U Test. (c) Analysis of the ARE activity in healthy VSMC as a function of 24h of AngII treatment. (d) Analysis of the ARE activity in AAA-SMC as a function of 24h of AngII treatment. (c and d) Statistical analysis was performed with ordinary one-way ANOVA testing for linear trend. Ns: not significant. The dotted line represents the trend line.

Supplementary Figure S2: ARE activity (RLU Nano-luc/Firefly) in individual cell cultures

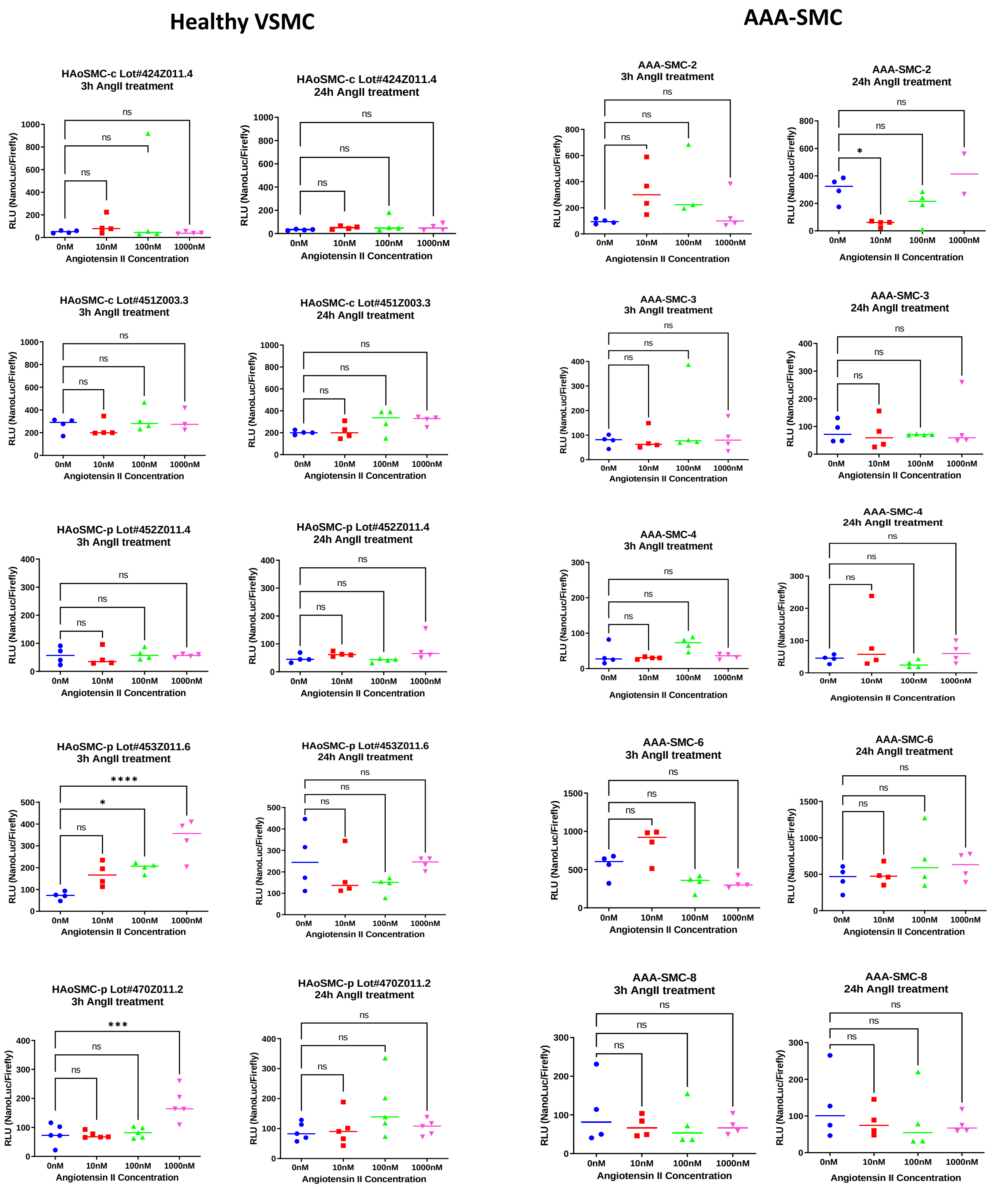

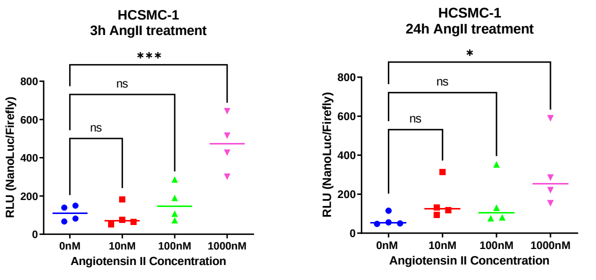

Supplementary Figure S2: ARE activity in individual healthy VSMC and AAA-SMC cultures after treatment for 3 and 24 h with 0 nM, 10 nM, 100nM and 1000 nM AngII. Cells were co-transfected with a reporter vector driving Nano-luc luciferase under the control of the anti-oxidative response element (ARE) and a reference plasmid driving firefly luciferase under the control of a constitutive promoter (SV40). Luminescence resulting from Nano-luc and Firefly luciferase was measured in four replicates for each cell culture and RLU was determined as light units (Nano-luc) / light units (Firefly) for each replicate. Data are sown with means of each quadruplicate measurement. Ordinary on-way ANOVA and Dunnett's multiple comparison test were used for statistical analysis

Supplementary Figure S3: ROS production in healthy VSMC and AAA-SMC.

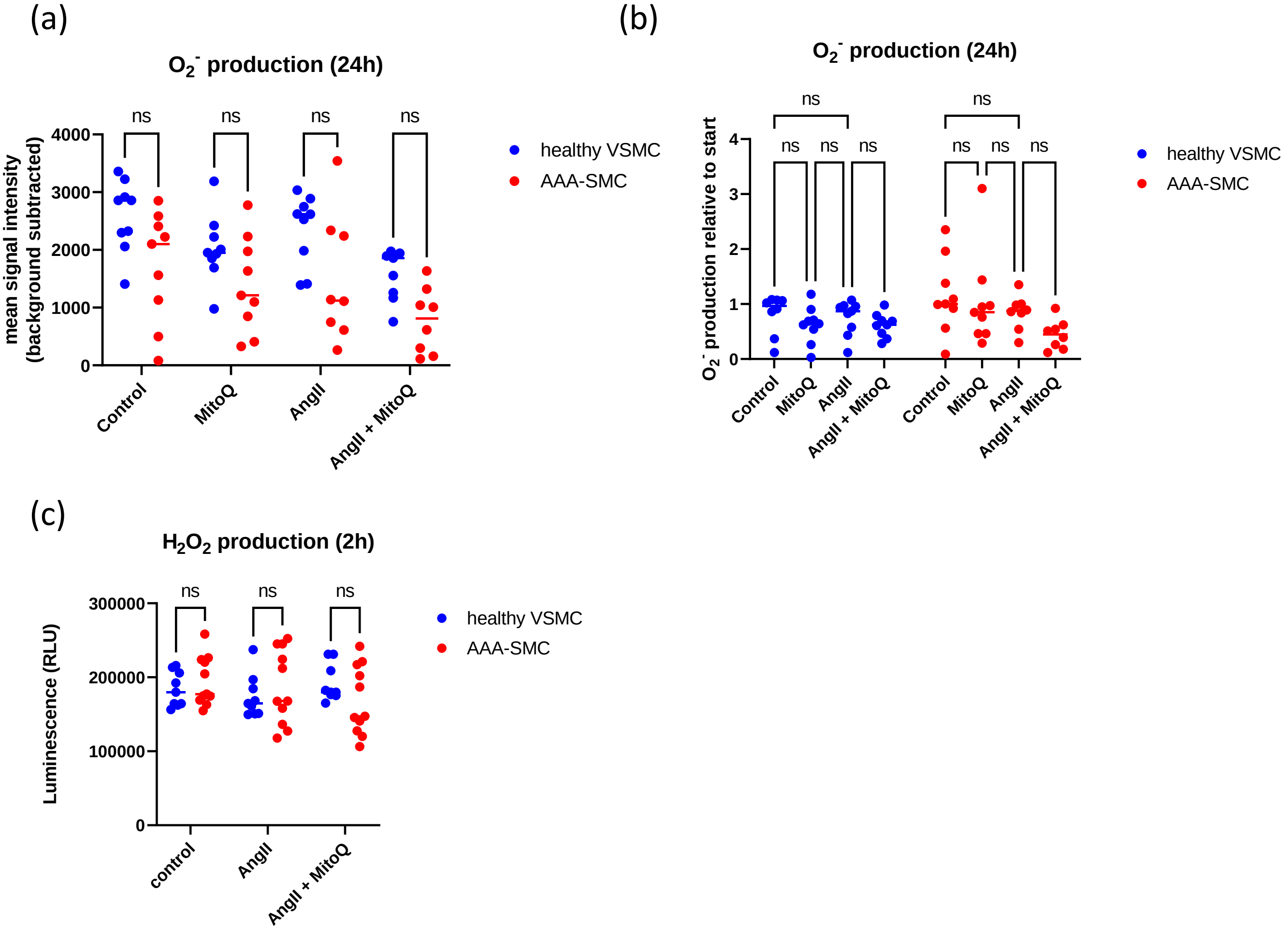

Supplementary Figure S3: ROS production in healthy VSMC and AAA-SMC. (a and b) Analysis of O_2^- production in controls and after 24 h of treatment with 100 nM MitoQ, or 1 µM AngII, or a combination of both. Cells were stained with CellROX green reagent and fluorescence was measured with a TECAN multiplate reader. Data are shown as mean signal intensity after background subtraction (a) or as absorbance relative to start (b) of three healthy and three AAA-SMC cultures analysed in triplicates. (c) Analysis of H_2O_2 amounts in controls and after 2 h of treatment with 1 µM AngII alone or in combination with 100 nM MitoQ. Detection of H_2O_2 was performed with a luminescent ROS-Glo H_2O_2 assay. Data are shown as relative light units (RLU) of three healthy and three AAA-SMC cultures analysed in triplicates and were analysed by 2way ANOVA and Šidák's multiple comparison test as a post-hoc test.

Supplementary Figure S4: Total NADPH/NADP+ levels in response to AngII.

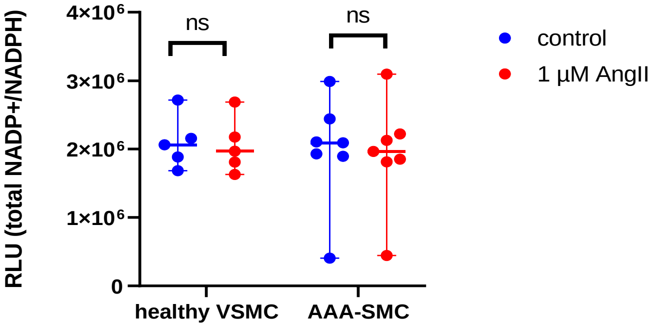

Supplementary Figure S4: Total NADPH/NADP+ levels in response to AngII. Healthy VSMC cultures (n = 5) and AAA-SMC cultures (n = 7) were seeded at a density of 8000 cells/well and treated with 1µM Angiotensin II (+ AngII) or left untreated (control) for 24h. Total NADPH/NADP+ was determined in triplicates by using a bioluminescent NADP/NADPH-Glo Assay as recommended by the manufacturer.

Supplementary Figure S5: DNA damage in response to Angiotensin II (3h)

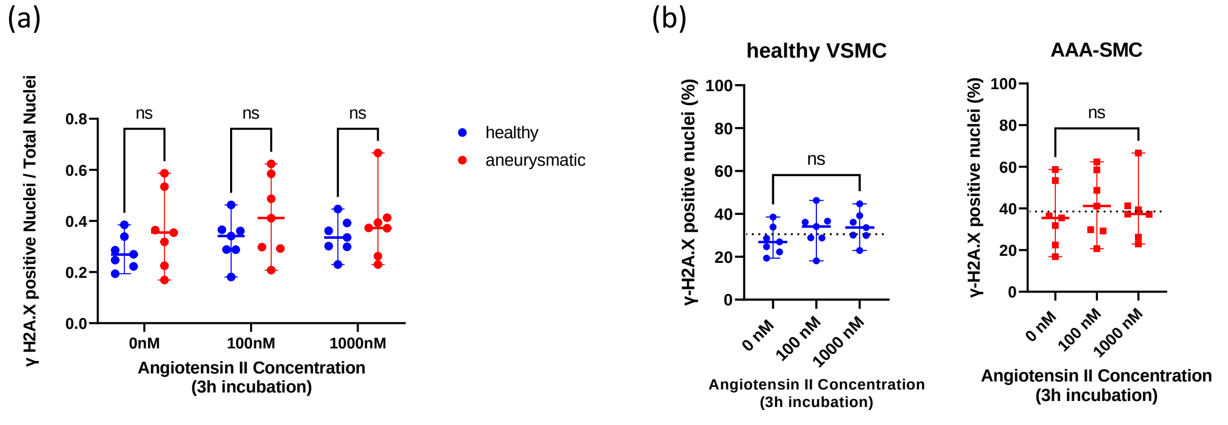

Supplementary Figure S5: DNA damage in response to Angiotensin II in AAA-SMC (n = 7 cultures) versus healthy VSMC (n = 7 cultures). Cells were incubated with increasing concentrations of Angiotensin II for 3h, fixed and immunostained with an anti-γ-H2A.X antibody as described in the Materials and Methods. Data points represent the mean percentage of γ-H2A.X -positive nuclei per total nuclei for each cell culture and are presented as median with range of n = 7 AAA-SMC cultures and n = 7 healthy VSMC cultures. Eight to fifteen images of each culture were evaluated for analysis. Statistical analysis was performed pairwise with Mann-Whitney U test. Ordinary one-way ANOVA was performed to test for a linear trend; ns = not significant .

Supplementary Figure S6: Viability of individual healthy VSMC cultures.

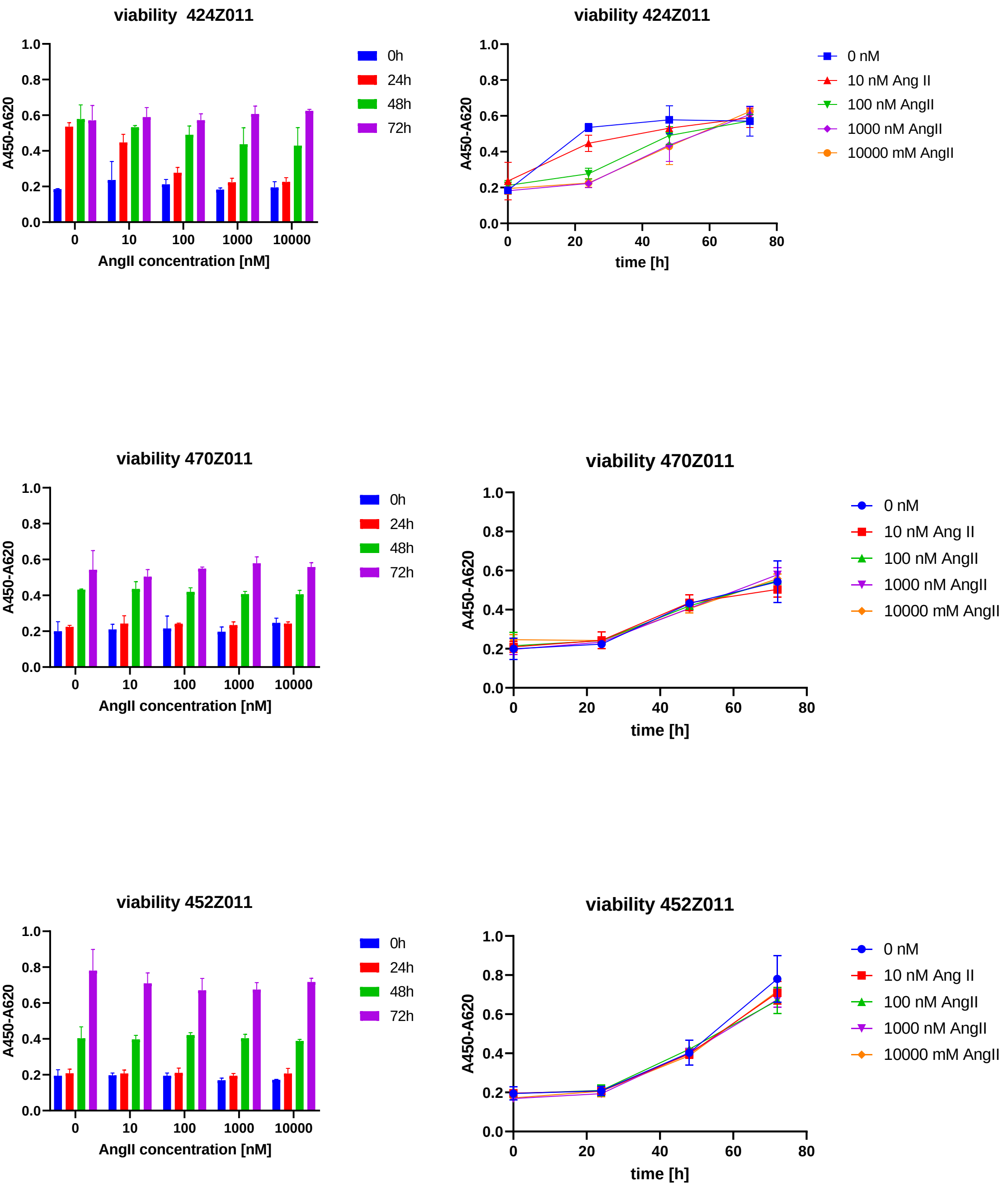

Supplementary Figure S6: Viability of individual healthy VSMC cultures. Cells were seeded at a density of 10000 cells/well in 96 well plates and serum deprived overnight before medium was changed to complete medium containing different concentrations of AngII. The medium was changed every 24 hours after AngII addition to avoid loss of AngII activity. WST-1 reagent was added at start and after 24, 48 and 72 hours, and absorbance was measured at 450 and 620 nm with a TECAN plate reader. Data show the difference between A450 and A620 at the indicated time points.

Supplementary Figure S7: Viability of individual AAA-SMC cultures.

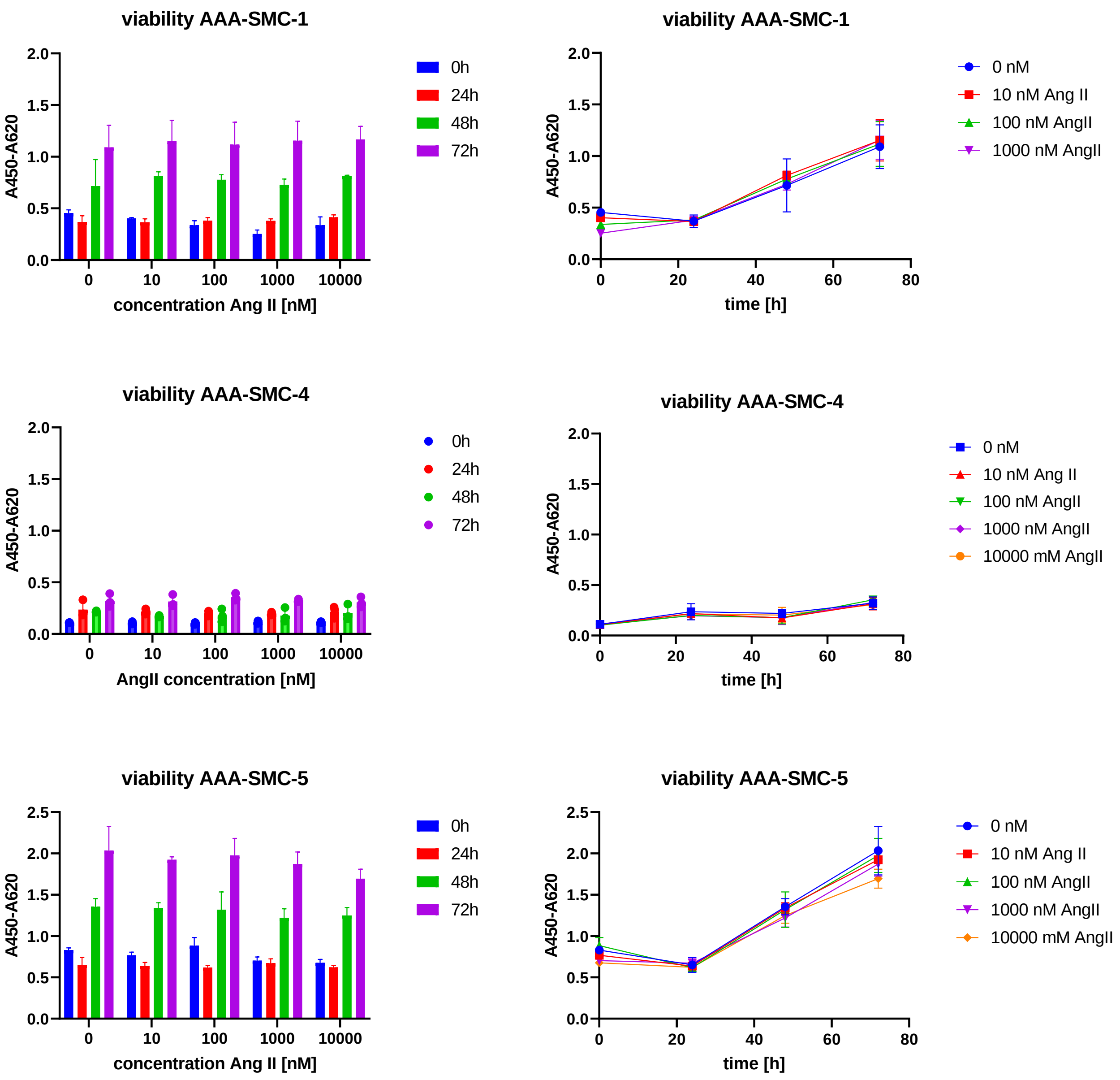

Supplementary Figure S7: Viability of individual AAA-SMC cultures. Cells were seeded at a density of 10000 cells/well in 96 well plates and serum deprived overnight before medium was changed to complete medium containing different concentrations of AngII. The medium was changed every 24 hours after AngII addition to avoid loss of AngII activity. WST-1 reagent was added at start and after 24, 48 and 72 hours, and absorbance was measured at 450 and 620 nm with a TECAN plate reader. Data show the difference between A450 and A620 at the indicated time points.
